# Susceptibility of Drug Resistant Hepatitis B Virus Mutants to Besifovir

**DOI:** 10.3390/biomedicines10071637

**Published:** 2022-07-07

**Authors:** Juhee Won, Ah Ram Lee, Mehrangiz Dezhbord, Da Rae Lee, Seong Ho Kim, Jong Chul Kim, Soree Park, Nayeon Kim, Byengjune Jae, Kyun-Hwan Kim

**Affiliations:** 1 Department of Pharmacology, School of Medicine, Konkuk University, Seoul 05029, Korea; 1wonjuhee@hanmail.net (J.W.); zoqlwns@naver.com (B.J.); 2 Department of Precision Medicine, School of Medicine, Sungkyunkwan University, Suwon 16419, Korea; ahram2g@naver.com (A.R.L.); m.dezhbord@yonsei.ac.kr (M.D.); edal_0422@naver.com (D.R.L.); seongho_0809@naver.com (S.H.K.); rlawhdcjf95@naver.com (J.C.K.); rhd37@naver.com (S.P.); michaela3310@naver.com (N.K.)

**Keywords:** hepatitis B virus, besifovir, reverse transcription, nucleos(t)ide analogue, drug resistance

## Abstract

Currently, interferon alpha and nucleos(t)ide analogues (NAs) are clinically available to treat hepatitis B virus (HBV) infection. Several NAs, including lamivudine (LMV), adefovir (ADV), entecavir (ETV) and tenofovir (TDF or TAF) have been approved and administered to chronic hepatitis B (CHB) patients. NAs inhibit HBV DNA synthesis by targeting the reverse transcriptase (RT) domain of HBV polymerase. Several mutations in the RT domain which lead to drug resistance against NAs have been reported, even for TDF and TAF which are highly potent with very low resistance rate. Besifovir (BFV) is a new antiviral dGMP analogue able to be used as a new NA drug for the control of CHB infection. Drug resistance to BFV is not well known due to its shorter duration of clinical use. Recently, we reported that rtL180M (M) and rtM204V (V) mutations, already resistant to LMV, are associated with BFV resistance. However, the susceptibility to BFV of previously known HBV mutants resistant to various drugs has not been studied. To investigate this, we performed in vitro drug susceptibility assays using natural and artificial mutants that are associated with resistance to LMV, ADV, ETV or TDF. As a result, LMV-resistant mutants were not susceptible to BFV and ETV-resistant clones showed partial resistance against BFV as well. However, ADV-resistant mutants were highly sensitive to BFV. In case of tenofovir-resistant mutations, the HBV mutants harboring primary mutations to tenofovir resistance were susceptible to BFV. Therefore, our study revealed that BSV may serve as an alternative drug for patients with ADV-, ETV-, TDF- or TAF-resistance.

## 1. Introduction

Globally, 292 million people are infected with hepatitis B virus (HBV) that can lead to chronic hepatitis, cirrhosis and hepatocellular carcinoma (HCC). Despite the development of vaccines and treatment, it remains a major concern worldwide [1].

Nucleos(t)ide analogues (NAs) compete with HBV polymerase and inhibit its replication by terminating DNA chain synthesis [2,3,4,5,6,7]. Specifically, NAs interfere with 3′ hydroxyl groups of deoxyribonucleic acid during elongation of HBV DNA, resulting in the failure of nascent DNA synthesis. Although, the NAs can suppress HBV DNA synthesis and alleviate symptoms, they cannot remove cccDNA, the persistent HBV minichromosome, and there are also drug usage limitations due to the occurrence of resistance by long-term therapy [8]. Several NAs, including lamivudine (LMV), adefovir (ADV) and entecavir (ETV), have been used for the treatment of HBV [9,10,11], however, emerging drug resistance seems inevitable [12,13].

Tenofovir (TFV), an active form of tenofovir disoproxil fumarate (TDF) and tenofovir alafenamide (TAF), is recommended as a first-line therapeutic option in recent HBV treatment guidelines because tenofovir resistance has not been found or appears with low probability [14,15]. Even though it has a high genetic barrier to resistance, we have previously reported a novel quadruple mutation (CYEI; rtS106C (C), rtH126Y (Y), rtD134E (E) and rtL269I (I)) that renders TFV resistance [16].

Since May 2017, besifovir (BFV), a new antiviral dGMP analogue, was approved as a new NA drug for treating naïve chronic hepatitis B (CHB) patients in South Korea [17]. BFV is an acyclic nucleotide phosphonate with a similar chemical structure to ADV and TDF [18,19,20].

BFV has similar clinical function as ETV or TDF including undetectable HBV DNA, ALT normalization or HBeAg seroconversion with the advantage of possessing lesser side effects such as osteoporosis or renal dysfunction [21,22,23].

We have previously reported that two major LMV-resistant mutations, rtL180M (M) and rtM204V (V), were associated with BFV resistance [24]. Therefore, additional investigations on BFV are required to select it as an alternative drug for patients with drug-resistance. Here we studied drug susceptibility of mutants isolated from patients with LMV-, ADV-, ETV- or TDF-resistance, and compared the results with the mutant clones constructed artificially in vitro.

## 2. Materials and Methods

### 2.1. Clinically Isolated HBV Mutant Clones and Construction of Artificial HBV Mutants

The patient-derived clone 50-2 and artificially constructed MV mutant (rtL180M + rtM204V) with resistance to LMV were described in our previous study [9]. HBV clones 10-16 and 10-17, which were isolated from serum of ADV-resistant patients, were reported previously [25]. Clinical isolates 69-2 and 71-3 from serum of ETV-resistant patients were reported in our preceding study [26]. The patient-derived tenofovir-resistant HBV mutant clones 1-1 and 1-13, and artificial mutants CYEI (rtS106C + rtH126Y + rtD134E + rtL269I) and CYELMVI (rtS106C + rtH126Y + rtD134E + rtV173L + rtL180M + rtM204V + rtL269I) were revealed in our previous study [16].

### 2.2. Cell Culture, Transfection and Drug Treatment

Human hepatoma cell line Huh7 was purchased from American Type Culture Collection (ATCC) and was cultured in Dulbecco’s modified Eagle’s medium (DMEM; welgene, Gyeongsan-si, Korea) supplemented with 10% fetal bovine serum (FBS; Capricorn Scientific, Ebsdorfergrund, Germany), and 1% penicillin/streptomycin (Gibco, Grand Island, NY, USA) at 37 °C in a 5% CO_2_ incubator. Approximately 9 × 10^5^ cells were seeded into 6-well plates. Sixteen hours after seeding, the replication-competent HBV 1.2mer clones, including wild type or other mutations, were transfected into the cells by Lipofectamine 2000. After 5 h, LMV (kindly provided by GlaxoSmith Kline, Brentford, UK), ADV (kindly provided by Gilead Science, Foster City, CA, USA), ETV (kindly provided by Bristol-Myers Squibb, New York, NY, USA), tenofovir (kindly provided by Dong-A Pharmaceutical Co., Seoul, Korea) or BFV (thoughtfully afforded by Ildong Pharmaceutical Co., Seoul, Korea) were administered daily at the indicated concentration with fresh medium. At 4 days post-transfection, supernatants in culture were collected and analyzed for levels of secreted HBV e antigens (HBeAg) by ELISA, and cells were harvested for Southern blot analysis to evaluate the HBV DNA replication.

### 2.3. ELISA

Culture supernatants were collected before harvesting cells. To confirm the transfection yield of HBV clones, the level of secreted HBeAg was measured by enzyme-linked immunosorbent assay (ELISA) using a kit (Wantai Pharm Inc., Beijing, China) in accordance with the manufacturer’s instructions. The culture supernatants were diluted 20-fold for HBeAg. At a wavelength of 450 nm, optical density (OD) values were measured using a spectrophotometer (SpectraMAX Plus 384).

### 2.4. Southern Blot

To determine the in vitro drug susceptibility and HBV replication of each mutant clone, Southern blot analysis was performed as previously described with some modifications [27]. Summary of the procedure is as follows: cells were harvested 4 days after transfection and lysed with HEPES buffer (10 mM HEPES at pH 7.5, 1 mM EDTA, 100 mM NaCl) containing 1% Np-40. To digest transfected plasmids, lysates were treated with nuclease buffer I containing 10 mM CaCl_2_, 12 mM MgCl_2_ and 10 units of DNase I (Roche, Mannheim, Germany) and were incubated at 37 °C for 2 h. Intracellular HBV capsids were then precipitated with 7.4% PEG (polyethylene glycol 8000, Sigma, St. Louis, MO, USA) and were incubated on ice overnight. To completely eliminate residual plasmids, the precipitates were digested with nuclease buffer II containing 10 mM Tris-HCl, 8 mM CaCl_2_, 6 mM MgCl_2_ and 10 units of DNase I at 37 °C for 20 min. After incubation, to release capsid-associated HBV DNA, the capsids were digested with 240 µg/mL proteinase K (Roche) in the presence of 0.5% sodium dodecyl sulfate (SDS) at 37 °C for 2 h, followed by phenol/chloroform/isoamyl alcohol (25:24:1) extraction. HBV DNA was then precipitated with 100% ethanol and sodium acetate. Total HBV DNA was separated by electrophoresis on 1% agarose (LE, analytical grade, Promega, Madison, WI, USA) gel and transferred onto a positively charged Hybond-XL membrane (GE Healthcare, Buckinghamshire, UK) by alkaline transfer method. An HBV probe containing 7 fragments of digoxigenin (DIG), which targets the whole genome, of 200–300 bp length was synthesized with a PCR DIG Probe Synthesis Kit (Roche) for detection of HBV DNA intermediates [28]. After hybridization with DIG-probe in Church buffer (0.5 M Na_2_HPO_4_ (pH 7.2), 1 mM EDTA, 7% SDS and 1% BSA), HBV intermediates were detected using a DIG Nucleic Acid Detection Kit (Roche) following the manufacturer’s instructions. HBV DNA was visualized by ImageQuant 800 (Amersham, Buckinghamshire, UK). The replication of HBV DNA was quantified by Multi-Gauge V3.2 software (Fujifilm, Tokyo, Japan).

### 2.5. Statistically Analysis

At least three independent experiments were performed for all analyses. Data are mean ± SD. Statistical significance was evaluated by one-way ANOVA in GraphPad Prism v6.

## 3. Results

### 3.1. The LMV-Resistant MV Mutant Is Resistant to BFV

**Figure 1:**
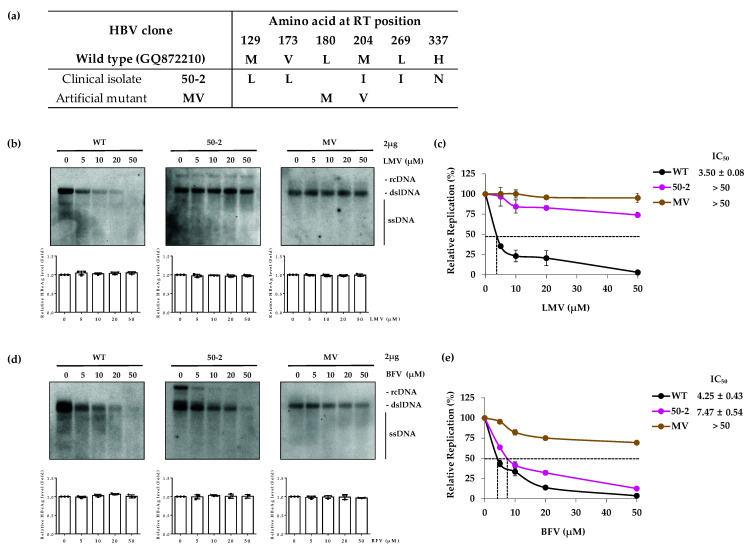
In vitro susceptibility of LMV-resistant HBV clones to BFV. (**a**) The sequences of LMV clones are shown. (**b**, top panels) HBV 1.2mer replicons harboring LMV resistance mutations in their RT domain were transfected into the Huh7 cells. The transfected cells were treated with LMV for 4 days before harvest for the drug susceptibility assay. The replication of HBV DNA was analyzed by Southern blot. (**c**) The IC_50_ values for patient-derived clone 50-2 and in vitro constructed clone MV were compared with WT. (**d**) Resistance of mutants to BFV was measure using Southern blot. (**e**) IC_50_ values for BFV were determined by Southern blot and were quantified by Multigauge software. (**b**,**d**, bottom panels) Secreted HBeAg was measured by ELISA. All data were obtained from at least three independent experiments (mean ± SD). LMV, lamivudine; BFV, besifovir; WT, wild type; HBeAg, HBV e antigen.

To evaluate the susceptibility of LMV-resistant HBV mutants to BFV, the patient-derived LMV-resistant clone 50-2 which harbors rtM129L + rtV173L + rtM204I + rtL269I + rtH337N mutations and the artificially constructed clone MV harboring rtL180M + rtM204V in the RT domain (Figure 1a) were selected. Initially to confirm the LMV resistance of the selected clone, the wild type (WT) and mutant clones (50-2 and MV) were transfected into the Huh7 cells and drug susceptibility was examined by Southern blot analysis. The level of secreted HBeAg was analyzed by ELISA to confirm the transfection yield (Figure 1b, bottom panels). Following LMV treatment, the WT HBV DNA levels were decreased in a dose-dependent manner. However, in the case of mutant clone 50-2, there were no considerable reductions in the HBV DNA intermediates after LMV treatment. Consistent with the data from patient-derived clones, the in vitro constructed LMV-resistant mutant clone MV showed no considerable decrease in level of HBV DNA after LMV treatment (Figure 1b, upper panels). The IC_50_ values for WT, 50-2 and MV were 3.50 ± 0.08, >50 and >50 μM, respectively (Figure 1c). The fold difference in IC_50_ values for 50-2 and MV compared to the WT were more than 14.2-fold, for both. These data confirmed that 50-2 and MV mutants were resistant to LMV, which is consistent with the previously obtained data [9,29,30].

Next, the BFV susceptibility assay using WT, 50-2 and MV mutants was performed. Besifovir dipivoxil maleate is an oral administrable drug that is hydrolyzed by esterase in liver and intestine and is converted into BFV (active metabolite, LB80317) by separating acetyl groups. Since there is no converting enzyme in vitro, a susceptibility assay was performed using BFV. The level of HBeAg was evaluated to confirm transfection yield (Figure 1d, bottom panels). While WT HBV DNA was decreased by BFV in a dose-dependent manner, clones 50-2 and MV showed strong resistance to BFV treatment (Figure 1d, upper panels). The IC_50_ values for WT, 50-2 and MV were 4.25 ± 0.43, 7.47 ± 0.54 and >50 μM, respectively (Figure 1e). The fold difference in IC_50_ values for 50-2 and MV relative to the WT were 1.8-fold and >11.8-fold higher. These data showed that the 50-2 clone was susceptible to BFV while the MV clone was also substantially resistant to the BFV.

### 3.2. The ADV-Resistant RT Mutants Are Susceptible to BFV

**Figure 2:**
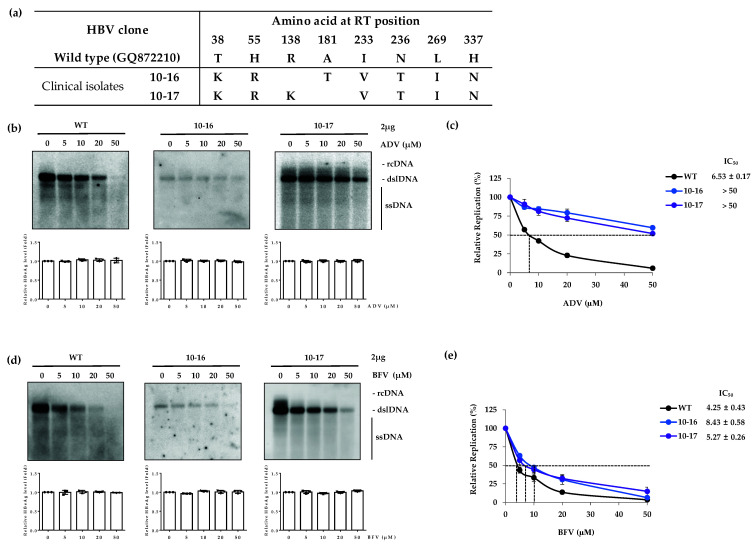
In vitro BFV susceptibility of ADV-resistant patient-derived clones. (**a**) Representative sequence of ADV-resistant clones derived from patients. (**b**, top) The ADV-resistant clinical isolates were transfected into the Huh7 cells, followed by administration with ADV for 4 days. The susceptibility to ADV was measured by Southern blot. (**c**) The IC_50_ values for ADV-resistant clones were compared to that of WT. (**d**, top panels) After treatment with BFV, the replication of ADV-resistant HBV RT mutants was analyzed by Southern blot. (**e**) The IC_50_ values of BFV for WT and mutant HBV clones were quantified and compared. (**b**,**d**, bottom panels) HBeAg in culture supernatant was measured by ELISA. The data were obtained from at least three independent experiments (mean ± SD). BFV, besifovir; ADV, adefovir.

To examine whether BFV reduces HBV DNA replication in ADV-resistant mutants, a susceptibility assay with ADV-resistant patient-derived mutant clones (10-16 and 10-17) was performed. The RT domain mutation profile of the 10-16 and 10-17 clones are summarized in Figure 2a. Among these mutations, the rtA181T, rtI233V and rtN236T are known as the major ADV-resistant mutations. To confirm the resistance of these clones to ADV, HBV clones (WT, 10-16 and 10-17) were transfected into the Huh7 cells and treated with ADV. The HBeAg level was examined by ELISA as a control for transfection yield (Figure 2b, bottom panels). The Southern blot results showed that the WT HBV was susceptible to ADV in a dose-dependent manner, whereas clones 10-16 and 10-17 were resistant to ADV (Figure 2b, upper panels). The IC_50_ values for WT, clone 10-16 and clone 10-17 were 6.53 ± 0.17, >50 and >50 μM, respectively (Figure 2c). The fold difference in IC_50_ values for 10-16 and 10-17 as compared to the WT were >7.6-fold for both mutants. Consistent with our previous data [25], these results confirmed that clones 10-16 and 10-17 are resistant to ADV. Susceptibilities to BFV were then evaluated with clones 10-16 and 10-17. All three tested clones (WT, 10-16 and 10-17) demonstrated susceptibility to BFV as the intracellular HBV capsid DNA levels were dramatically reduced by BFV treatment (Figure 2d). The IC_50_ values for WT, 10-16 and 10-17 were 4.25 ± 0.43, 8.43 ± 0.58 and 5.27 ± 0.26 μM, respectively (Figure 2e). The fold difference in IC_50_ for 10-16 and 10-17 were 2.0-fold and 1.2-fold higher than that of WT, respectively. Therefore, BFV efficiently suppressed the replication of ADV-resistant RT mutants.

### 3.3. The ETV-Resistant RT Mutants Are partially Resistant to BFV

**Figure 3:**
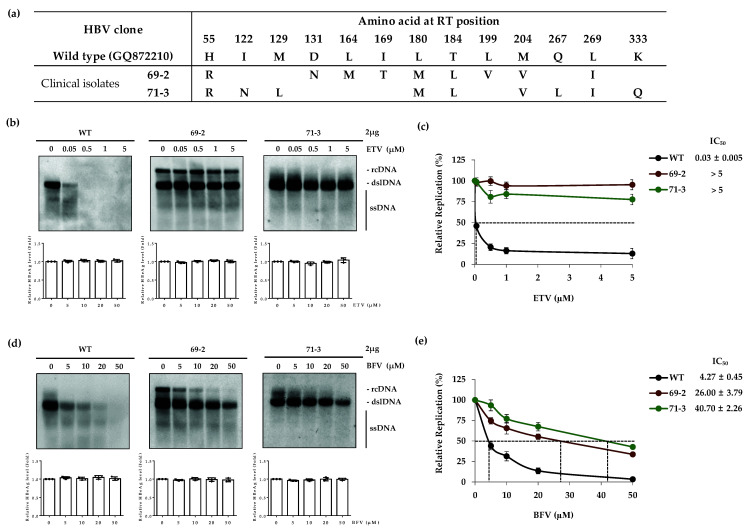
In vitro BFV susceptibility assay for ETV-resistant HBV clones. (**a**) The sequence of clinical isolates with ETV-resistant mutations. (**b**, top panels) To analyze the susceptibility of mutants to ADV, clones were transfected into the Huh7 cells and ETV was treated for 4 days in a dose-dependent manner. HBV replication level was measured by Southern blot. (**c**) The IC_50_ values for patient-derived clones were compared to WT. (**d**, top panels) The HBV DNA replication of ETV-resistant clones, treated with BFV for 4 days, was determined by Southern blot. (**e**) The IC_50_ of BFV for WT and ETV-resistant HBV clones. (**b**,**d**, bottom panels) Secreted HBeAg into supernatant was assessed. Data in (**b**–**e**) were obtained from at least three independent experiments (mean ± SD). BFV, besifovir; ETV, entecavir.

Next, we seek to know the susceptibility of patient-derived ETV-resistant clones (69-2, 71-3) to BFV in vitro. As shown in Figure 3a, clone 69-2 has rtH55R, rtD131N, rtL164M, rtI169T, rtL180M, rtT184L, rtL199V, rtM204V and rtL269L mutations.

Clone 71-3 harbors rtH55R, rtI122F, rtL180M, rtT184L, rtM204V, rtQ267L, rtL269I and K333Q substitutions in the RT domain. The rtI169T, rtL180M and rtT184L mutations are important for resistance to ETV [26]. For the drug susceptibility assay, the WT, clone 69-2 or 71-3 were transfected into the Huh7 cells. Transfection yield was confirmed with equal levels of secreted HBeAg (Figure 3b, bottom panels). While the replication of HBV WT was considerably decreased by ETV in a dose-dependent manner, clones 69-2 and 71-3 showed significant resistance as expected. The IC_50_ values for WT, clone 69-2 and clone 71-3 were 0.03 ± 0.005, >5 and >5 μM, respectively (Figure 3c). The fold difference in IC_50_ values for 69-2 and 71-3 were >166.7-fold compared to the WT.

In the susceptibility assay with BFV, while WT HBV DNA levels decreased after BFV treatment, the mutant clones 69-2 and 71-3 exhibited resistance to BFV (Figure 3d). IC_50_ values for WT, 69-2 and 71-3 were 4.25 ± 0.43, 26.00 ± 3.79 and 40.70 ± 2.26 μM, respectively (Figure 3e). The fold difference in IC_50_ values for 69-2 and 71-3 were 6.1-fold and 9.6-fold higher than the WT, respectively. Interestingly, despite the existence of MV mutation in the RT domain of clones 69-2 and 71-3, the level of their resistance to BFV was considerably lesser than the artificially constructed clone counterpart harboring MV mutation which were highly resistant (IC_50_ > 50 μM) (Figure 1d). Therefore, these results suggest that clones 69-2 and 71-3 (derived from ETV resistant patients) were partially resistant to BFV.

### 3.4. The HBV Mutants Harboring Primary Mutations to TFV Resistance Are Susceptible to BFV

**Figure 4:**
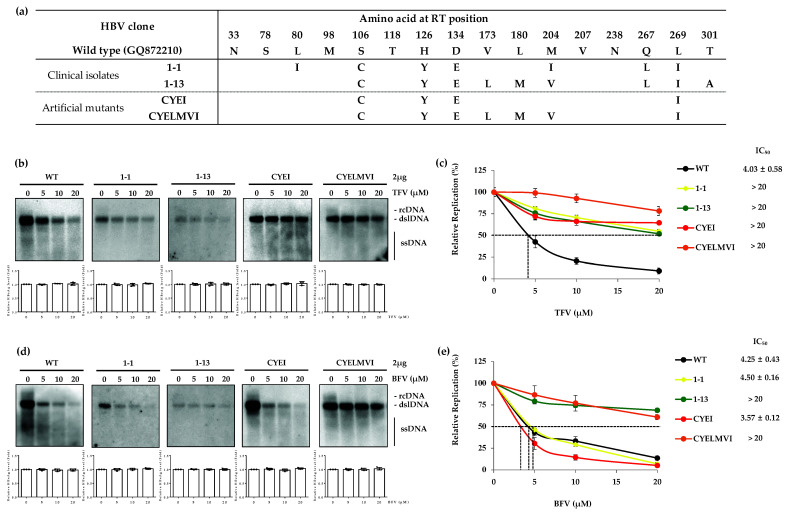
In vitro susceptibility of TFV-resistant HBV clones to BFV. (**a**) The sequence information of patient-derived or artificial HBV mutant clones which are resistant to TFV. (**b**, top panels) The patient-derived clones or artificial mutants were transfected into the Huh7 cells. TFV was then administered for 4 days at the indicated concentrations. The level of HBV replication was analyzed by Southern blot. (**c**) The IC_50_ value for TFV-resistant clones and WT were compared. (**d**, top panels) The drug susceptibility for BFV were determined by Southern blot analysis. (**e**) The IC_50_ values of clinical isolates or artificial constructs were compared with WT. (**b**,**d**, bottom panels) The transfection yield was confirmed by measuring the level of HBeAg in the culture supernatant using ELISA. The data were obtained from at least three independent experiments (mean ± SD). BFV, besifovir; TFV, tenofovir.

In order to explore the drug susceptibility of TFV-resistant HBV mutants to BFV, TFV-resistant patient-derived clones (1-1 and 1-13) and artificially constructed mutants (CYEI and CYELMVI) were selected [16]. As shown in Figure 4a, the clone 1-1 had an RT domain harboring the rtL80I, rtC106S, rtH126Y, rtD134E, rtM204I and rtL269I substitutions.

The 1-13 clone carried rtS106C, rtH126Y, rtD134E, rtV173L, rtL180M, rtM204V, rtQ267L, rtL269I and rtT301A mutations within its RT domain. For TFV susceptibility assay, the replication levels of WT and mutant HBV capsid DNA were measured in Huh7 cells. As expected, TFV-resistant HBV mutant clones were not susceptible to TFV while replication of WT HBV DNA was significantly reduced in a dose-dependent manner (Figure 4b). The IC_50_ values for WT, 1-1, 1-13, CYEI and CYELMVI clones were 4.03 ± 0.58, >20, >20, >20, and >20 μM, respectively (Figure 4c). The fold differences in IC_50_ values for 1-1, 1-13, CYEI and CYELMVI relative to the WT were > 5.0-fold, respectively. Lastly, the BFV susceptibility assay was performed. The 1-1 and CYEI mutants and the WT clones showed high susceptibility to BFV. However, as expected, clones 1-13 and CYELMVI, which harbor MV mutations, were highly resistant to BFV treatment (Figure 4d). IC_50_ values for WT, 1-1, 1-13, CYEI and CYELMVI were 4.25 ± 0.43, 4.50 ± 0.16, >20, 3.57 ± 0.12 and >20 μM, respectively (Figure 4e). The fold difference in IC_50_ values for 1-1, 1-13, CYEI, and CYELMVI as compared to the WT were 1.1-fold, >4.7-fold, 0.8-fold and >4.7-fold, respectively. Thus, the TFV-resistant mutant clones 1-1 and CYEI were considerably susceptible to BFV, but MV-harboring mutant clones 1-13 and CYELMVI were resistant to BFV.

## 4. Discussion

BFV, recently (2017) approved as an anti-HBV drug in South Korea, is a relatively new antiviral dGMP analogue [17]. BFV is an acyclic nucleotide phosphonate with a similar chemical structure to ADV and TFV [31,32]. BFV resistance has not been reported thus far due to its short usage period. However, recently we revealed that rtL180M and rtM204V, which are associated WITH LMV resistance, are not susceptible to BFV [24]. Here we further identified the BFV susceptibility of already resistant HBV mutants including the mutants associated with LMV, ADV, ETV and TDF resistance. The IC_50_ values (and fold resistance) of all of HBV RT mutation clones which were used in this study are summarized in Table 1.

In our study, the LMV-resistant 50-2 and MV mutant clones showed strong resistance to BFV. Interestingly, despite clone 50-2 having rtM204I, instead of rtM204V, along with rtL180M mutation, it did not show resistance to BFV. Clone 50-2 harbors additional mutations including rtM129L, rtV173L, rtL269I and rtH337N. As the results are the same, the rtM204I substitution was considerably susceptible to BFV, as shown in the 1-1 clone (Figure 4d). The rtL236I and rtH337N mutations in the RT domain of ADV-resistant HBV clones 10-16 and 10-17 did not confer any resistance to BFV (Figure 2b). The third RT mutation, rtV173L [33], which appeared in LMV-resistant patients, did not influence resistance to BFV.

The in vitro drug susceptibility assay for the ADV-resistant HBV clones 10-16 and 10-17 showed that BFV effectively suppressed their replication levels to the same level of reduction as in WT clone (Figure 2b). These results show that rtT38K, rtH55R, rtR138K, rtI233V, rtN238T, rtL269I, rtN337H, especially rtA181T and rtN236T, as major mutations (Figure 2a) for ADV resistance, are not associated with resistance to BFV and are equally susceptible to BFV as are WT.

The previously identified ETV-resistant HBV clones 69-2 and 71-3 harboring rtL180M and rtM204V substitutions, which are responsible for cross-resistance to LMV and ETV [29,30,34], were partially affected by BFV. Intriguingly, although these clones showed high fold difference in IC_50_ values compared to the WT, the fold differences were lower than those clones with only rtL180M or rtM204V mutations. The artificially constructed mutant which had only MV variation, was almost unaffected by BFV treatment (Figure 1). Moreover, there are other mutations in the RT domains of these clones that include rtH55R, rtD131N, rtL164M, rtI169T, rtT184L, rtL199V and rtL269I for 69-2 and rtH55R, rtM129L, rtT184L, rtQ267L, rtL269I and rtK333Q for 71-3 (Figure 3a). One possibility is that the combination of some mutations with MV may change the structure of the BFV attachment site which partially blocks its binding. Further study is required to reveal the mutations involved in the resistance of MV harboring clones.

Analysis of resistance to BFV for TFV-resistant HBV clones showed that the patient-derived clone 1-1 was considerably susceptible to BFV while the clone 1-13 showed low drug susceptibility. The BFV resistance of clone 1-13 is thought to be due to its rtL180M and rtM204V mutations. The susceptibility assay was not performed for telbivudine (LdT) in this study. However, clone 1-1 has rtL80I and rtM204I mutations which were reported to confer resistance to LdT [35]. The high sensitivity of clone 1-1 to BFV makes one expect that BFV may successfully decrease the HBV DNA replication of LdT-resistant mutants. We have previously reported that the in vitro constructed clone CYEI is a mutant harboring a quadruple mutation that is associated with TFV resistance [16]. This clone was highly sensitive to BFV, similarly to WT (Figure 4e). This data is in line with our finding regarding clone 10-16 harboring the rtL269I mutation that did not show BFV-resistance. (Figure 2). In the BFV susceptibility assay for the mutants with CYELMVI variation, as expected, BFV could not efficiently suppress HBV DNA replication (Figure 4e) due to the existence of the MV mutation in this mutant clone (Figure 1 and Figure 3). Based on the results obtained here, the ETV-resistant clones 69-2 and 71-3 were somewhat resistant to BFV but to a lesser extent as compared to the MV harboring mutants (Figure 1d). These data suggest that BFV cannot efficiently repress the HBV DNA replication of TFV-resistant mutants unless the rtL180M and rtM204V mutations are not incorporated. Results for LMV-resistant clones showed that if there are no MV mutations (clone 50-2), BFV could be effective. This effect of BFV is consistent with previous data [36]. TFV-resistant mutations were also susceptible to BFV in the absence of MV mutations. These results suggest further study to reveal whether the mutations, which are already known to occur during antiviral treatment with other NAs, are associated with resistance to BFV. Additionally, the ETV-resistant mutations which decreased the resistance of MV variation to BFV mutations propose the existence of mutations which could counteract resistance to NAs.

ETV and TFV have been recommended as the first-line therapeutic options for CHB patients in practice guidelines [14,15,37]. BFV is a new strong antiviral NA which has low toxicity and a high barrier to resistance [38]. Previous reports described that BFV is effective and safe in long-term treatment of naïve and TFV-experienced patients. Moreover, this antiviral reagent showed better safety, tolerance and efficacy than TFV [18,23,39].

In this study we raised the question whether BFV can be selected as an alternative drug to control the HBV mutants which revealed resistance against common NAs including LMV, ADV, ETV or TFV.

## 5. Conclusions

Overall, the susceptibility of drug resistant mutant clones to BFV were evaluated in the current study. Accordingly, our results suggest that BFV can be used as an alternative drug for patients with drug-resistance to ADV and TFV as long as the HBV mutants do not include the MV sequence. This study could be beneficial when assigning clinical guidelines for BFV treatment of CHB patients.

## Figures and Tables

**Table 1:** Summary of in vitro IC_50_ values of HBV clones to NAs.

Drug	Clone	IC_50_ (µM) (Fold Resistance)				
Resistance		BFV	LMV	ADV	ETV	TFV
	WT	4.25 ± 0.43 (1)	3.50 ± 0.08 (1)	6.53 ± 0.17 (1)	0.03 ± 0.005 (1)	4.03 ± 0.58 (1)
LMV/CLV/ETV	50-2	7.47 ± 0.54 (1.8)	>50 (>14.2)			
	MV	>50 (>11.8)	>50 (>14.2)			
ADV	10-16	8.43 ± 0.58 (2.0)		>50 (>7.6)		
	10-17	5.27 ± 0.26 (1.2)		>50 (>7.6)		
ETV	69-2	26.00 ± 3.79 (6.1)			>5 (>166.7)	
	71-3	40.70 ± 2.26 (9.6)			>5 (>166.7)	
TFV	1-1	4.50 ± 0.16 (1.1)				>20
	1-13	>20 (>4.7)				>20
	CYEI	3.57 ± 0.12 (0.8)				>20
	CYELMVI	>20 (>4.7)				>20

BFV, besifovir; LMV, lamivudine; ADV, adefovir; ETV, entecavir; TFV, tenofovir; CLV, clevudine.

## Data Availability

Not applicable.

## References

[1] Nguyen M.H., Wong G., Gane E., Kao J.H., Dusheiko G. (2020). Hepatitis B Virus: Advances in Prevention, Diagnosis, and Therapy. Clin. Microbiol. Rev..

[2] Tu T., Budzinska M.A., Vondran F.W.R., Shackel N.A., Urban S. (2018). Hepatitis B Virus DNA Integration Occurs Early in the Viral Life Cycle in an In Vitro Infection Model via Sodium Taurocholate Cotransporting Polypeptide-Dependent Uptake of Enveloped Virus Particles. J. Virol..

[3] Ko C., Chakraborty A., Chou W.M., Hasreiter J., Wettengel J.M., Stadler D., Bester R., Asen T., Zhang K., Wisskirchen K. (2018). Hepatitis B virus genome recycling and de novo secondary infection events maintain stable cccDNA levels. J. Hepatol..

[4] Dusseaux M., Masse-Ranson G., Darche S., Ahodantin J., Li Y., Fiquet O., Beaumont E., Moreau P., Riviere L., Neuveut C. (2017). Viral Load Affects the Immune Response to HBV in Mice With Humanized Immune System and Liver. Gastroenterology.

[5] Konig A., Yang J., Jo E., Park K.H.P., Kim H., Than T.T., Song X., Qi X., Dai X., Park S. (2019). Efficient long-term amplification of hepatitis B virus isolates after infection of slow proliferating HepG2-NTCP cells. J. Hepatol..

[6] Yasutake Y., Hattori S.I., Tamura N., Matsuda K., Kohgo S., Maeda K., Mitsuya H. (2020). Structural features in common of HBV and HIV-1 resistance against chirally-distinct nucleoside analogues entecavir and lamivudine. Sci. Rep..

[7] Seifer M., Patty A., Serra I., Li B., Standring D.N. (2009). Telbivudine, a nucleoside analog inhibitor of HBV polymerase, has a different in vitro cross-resistance profile than the nucleotide analog inhibitors adefovir and tenofovir. Antivir. Res..

[8] Lopatin U. (2019). Drugs in the Pipeline for HBV. Clin. Liver Dis..

[9] Kwon S.Y., Park Y.K., Ahn S.H., Cho E.S., Choe W.H., Lee C.H., Kim B.K., Ko S.Y., Choi H.S., Park E.S. (2010). Identification and characterization of clevudine-resistant mutants of hepatitis B virus isolated from chronic hepatitis B patients. J. Virol..

[10] Hoofnagle J.H., Doo E., Liang T.J., Fleischer R., Lok A.S. (2007). Management of hepatitis B: Summary of a clinical research workshop. Hepatology.

[11] Kim J.H., Park Y.K., Park E.S., Kim K.H. (2014). Molecular diagnosis and treatment of drug-resistant hepatitis B virus. World J. Gastroenterol..

[12] Lai C.L., Dienstag J., Schiff E., Leung N.W., Atkins M., Hunt C., Brown N., Woessner M., Boehme R., Condreay L. (2003). Prevalence and clinical correlates of YMDD variants during lamivudine therapy for patients with chronic hepatitis B. Clin. Infect. Dis. Off. Publ. Infect. Dis. Soc. Am..

[13] Yuen M.F., Seto W.K., Chow D.H., Tsui K., Wong D.K., Ngai V.W., Wong B.C., Fung J., Yuen J.C., Lai C.L. (2007). Long-term lamivudine therapy reduces the risk of long-term complications of chronic hepatitis B infection even in patients without advanced disease. Antivir. Ther..

[14] European Association for the Study of the Liver (2017). EASL 2017 Clinical Practice Guidelines on the management of hepatitis B virus infection. J. Hepatol..

[15] Terrault N.A., Lok A.S.F., McMahon B.J., Chang K.M., Hwang J.P., Jonas M.M., Brown R.S., Bzowej N.H., Wong J.B. (2018). Update on prevention, diagnosis, and treatment of chronic hepatitis B: AASLD 2018 hepatitis B guidance. Hepatology.

[16] Park E.S., Lee A.R., Kim D.H., Lee J.H., Yoo J.J., Ahn S.H., Sim H., Park S., Kang H.S., Won J. (2019). Identification of a quadruple mutation that confers tenofovir resistance in chronic hepatitis B patients. J. Hepatol..

[17] Fung J., Lai C.L., Yuen M.F. (2008). LB80380: A promising new drug for the treatment of chronic hepatitis B. Expert Opin. Investig. Drugs.

[18] Ahn S.H., Kim W., Jung Y.K., Yang J.M., Jang J.Y., Kweon Y.O., Cho Y.K., Kim Y.J., Hong G.Y., Kim D.J. (2019). Efficacy and Safety of Besifovir Dipivoxil Maleate Compared With Tenofovir Disoproxil Fumarate in Treatment of Chronic Hepatitis B Virus Infection. Clin. Gastroenterol. Hepatol..

[19] Lai C.L., Ahn S.H., Lee K.S., Um S.H., Cho M., Yoon S.K., Lee J.W., Park N.H., Kweon Y.O., Sohn J.H. (2014). Phase IIb multicentred randomised trial of besifovir (LB80380) versus entecavir in Asian patients with chronic hepatitis B. Gut.

[20] Jung J.A., Kim S.R., Kim T.E., Kim J.R., Lee S.Y., Huh W., Ko J.W. (2012). Pharmacokinetic comparison of the maleate and free base formulations of LB80380, a novel nucleotide analog, in healthy male volunteers. Int. J. Clin. Pharmacol. Ther..

[21] Yuen M.F., Kim J., Kim C.R., Ngai V., Yuen J.C., Min C., Kang H.M., Shin B.S., Yoo S.D., Lai C.L. (2006). A randomized placebo-controlled, dose-finding study of oral LB80380 in HBeAg-positive patients with chronic hepatitis B. Antivir. Ther..

[22] Korean Association for the Study of the Liver (2019). KASL clinical practice guidelines for management of chronic hepatitis B. Clin. Mol. Hepatol..

[23] Song D.S., Kim W., Ahn S.H., Yim H.J., Jang J.Y., Kweon Y.O., Cho Y.K., Kim Y.J., Hong G.Y., Kim D.J. (2021). Continuing besifovir dipivoxil maleate versus switching from tenofovir disoproxil fumarate for treatment of chronic hepatitis B: Results of 192-week phase 3 trial. Clin. Mol. Hepatol..

[24] Kim J.C., Lee H.Y., Lee A.R., Dezhbord M., Lee D.R., Kim S.H., Won J., Park S., Kim N.Y., Shin J.J. (2022). Identification and Characterization of Besifovir-Resistant Hepatitis B Virus Isolated from a Chronic Hepatitis B Patient. Biomedicines.

[25] Ahn S.H., Park Y.K., Park E.S., Kim J.H., Kim D.H., Lim K.H., Jang M.S., Choe W.H., Ko S.Y., Sung I.K. (2014). The impact of the hepatitis B virus polymerase rtA181T mutation on replication and drug resistance is potentially affected by overlapping changes in surface gene. J. Virol..

[26] Park S., Park E.S., Koo J.E., Park Y.K., Lee A.R., Dezhbord M., Cho E.S., Ahn S.H., Kim D.H., Lee J.H. (2020). Entecavir-resistant hepatitis B virus decreases surface antigenicity: A full genome and functional characterization. Liver Int..

[27] Lee A.R., Cho J.Y., Kim J.C., Dezhbord M., Choo S.Y., Ahn C.H., Kim N.Y., Shin J.J., Park S., Park E.S. (2021). Distinctive HBV Replication Capacity and Susceptibility to Tenofovir Induced by a Polymerase Point Mutation in Hepatoma Cell Lines and Primary Human Hepatocytes. Int. J. Mol. Sci..

[28] Zoulim F., Locarnini S. (2009). Hepatitis B virus resistance to nucleos(t)ide analogues. Gastroenterology.

[29] Nakajima S., Watashi K., Kato T., Muramatsu M., Wakita T., Tamura N., Hattori S.I., Maeda K., Mitsuya H., Yasutake Y. (2021). Biochemical and Structural Properties of Entecavir-Resistant Hepatitis B Virus Polymerase with L180M/M204V Mutations. J. Virol..

[30] Wang Y., Liu S., Chen Y.U., Zheng S., Zhou L.I., Lu F., Duan Z. (2016). Lamivudine-resistant rtL180M and rtM204I/V are persistently dominant during combination rescue therapy with entecavir and adefovir for hepatitis B. Exp. Ther. Med..

[31] Lin C.L., Yang H.C., Kao J.H. (2016). Hepatitis B virus: New therapeutic perspectives. Liver Int. Off. J. Int. Assoc. Study Liver.

[32] Scruggs E.R., Dirks Naylor A.J. (2008). Mechanisms of zidovudine-induced mitochondrial toxicity and myopathy. Pharmacology.

[33] Delaney W.E.T., Yang H., Westland C.E., Das K., Arnold E., Gibbs C.S., Miller M.D., Xiong S. (2003). The hepatitis B virus polymerase mutation rtV173L is selected during lamivudine therapy and enhances viral replication in vitro. J. Virol..

[34] Pal A., Sarkar N., Saha D., Guha S.K., Saha B., Chakrabarti S., Chakravarty R. (2015). High incidence of lamivudine-resistance-associated vaccine-escape HBV mutants among HIV-coinfected patients on prolonged antiretroviral therapy. Antivir. Ther..

[35] Yin F., Wu Z., Fang W., Wu C., Rayner S., Han M., Deng F., Du R., Liu J., Wang M. (2015). Resistant mutations and quasispecies complexity of hepatitis B virus during telbivudine treatment. J. Gen. Virol..

[36] Yuen M.F., Han K.H., Um S.H., Yoon S.K., Kim H.R., Kim J., Kim C.R., Lai C.L. (2010). Antiviral activity and safety of LB80380 in hepatitis B e antigen-positive chronic hepatitis B patients with lamivudine-resistant disease. Hepatology.

[37] Kim K.H., Kim N.D., Seong B.L. (2010). Discovery and development of anti-HBV agents and their resistance. Molecules.

[38] Song J.E., Park J.Y. (2021). Besifovir dipivoxil maleate: A novel antiviral agent with low toxicity and high genetic barriers for chronic hepatitis B. Expert Opin. Pharmacother..

[39] Yim H.J., Kim W., Ahn S.H., Yang J.M., Jang J.Y., Kweon Y.O., Cho Y.K., Kim Y.J., Hong G.Y., Kim D.J. (2020). Besifovir Dipivoxil Maleate 144-Week Treatment of Chronic Hepatitis B: An Open-Label Extensional Study of a Phase 3 Trial. Am. J. Gastroenterol..

